# Integration of HIV pre-exposure prophylaxis (PrEP) services for pregnant and breastfeeding women in eight primary care clinics: results of an implementation science study

**DOI:** 10.21203/rs.3.rs-3648622/v1

**Published:** 2023-11-23

**Authors:** Aurelie Nelson, Kalisha Bheemraj, Sarah Schoetz Dean, Alex de Voux, Lerato Hlatshwayo, Rufaro Mvududu, Natacha Berkowitz, Caroline Neumuller, Shahida Jacobs, Stephanie Fourie, Thomas Coates, Linda-Gail Bekker, Landon Myer, Dvora Joseph Davey

**Affiliations:** Division of Epidemiology & Biostatistics, School of Public Health, University of Cape Town; Division of Epidemiology & Biostatistics, School of Public Health, University of Cape Town; Division of Infectious Diseases, Geffen School of Medicine, University of California Los Angeles; Division of Epidemiology & Biostatistics, School of Public Health, University of Cape Town; Division of Epidemiology & Biostatistics, School of Public Health, University of Cape Town; Division of Epidemiology & Biostatistics, School of Public Health, University of Cape Town; City of Cape Town Health, City of Cape Town; City of Cape Town Health, City of Cape Town; Western Cape Department of Health and Wellness, Metro Health Services,; Western Cape Department of Health and Wellness, Metro Health Services,; Division of Infectious Diseases, Geffen School of Medicine, University of California Los Angeles; Desmond Tutu Health Foundation; Division of Epidemiology & Biostatistics, School of Public Health, University of Cape Town; Division of Infectious Diseases, Geffen School of Medicine, University of California Los Angeles

**Keywords:** Pre-exposure prophylaxis, HIV, maternal and child health, implementation science, vertical transmission

## Abstract

**Background:**

Although HIV vertical transmission (VT) has declined significantly in sub-Saharan Africa, incident HIV infection in pregnant and postpartum women is estimated to account for roughly one-third of VT. Oral pre-exposure prophylaxis (PrEP) for pregnant and breastfeeding women (PBFW) is part of the recommended guidelines in South Africa since 2021; however, integration of PrEP services within antenatal (ANC) and postnatal care (PNC) remains limited.

**Methods:**

Between March 2022 and September 2023, we evaluated the acceptability, feasibility and sustainability of integrating PrEP for PBFW in high-HIV prevalence clinics after training and mentoring health care providers (HCP). We used the Reach Effectiveness-Adoption Implementation Maintenance (RE-AIM) framework to evaluate the intervention. Acceptability and maintenance were defined as the proportion of PBFW without HIV who initiated PrEP and the proportion of women continuing PrEP at 3 months in ANC or PNC services. Feasibility was defined as the proportion of trained HCPs (HIV lay counsellors and nurses/ midwives) who provided PrEP according to national guidelines, measured through post-training surveys and in-service assessments. Sustainability was defined as number of facilities and providers that continued to provide PrEP for PBFW past the mentoring period.

**Results:**

In 8 facilities providing ANC and PNC, we trained 224 HCP (127 nurses and 37 counsellors). Of those, we mentored 60 nurses, midwives and HIV counsellors working with PBFW, with 72% of nurse/midwives and 65% of counsellors scoring over 8/10 on the final mentoring assessment Overall, 12% (1493/12,614) of HIV-negative pregnant women started PrEP and 41% of those continued PrEP at 3-months. Among the HIV-negative breastfeeding women in postnatal care, 179/1315 (14%) initiated PrEP and 25% continued PrEP at 3-months. All 8 facilities continued providing PrEP 3-months after handover of the clinics.

**Conclusion:**

Integration of PrEP services in ANC and services for breastfeeding women was feasible, acceptable and sustainable. Acceptability and PrEP continuation showed improvement over time. Barriers to the PrEP integration were observed including the lack of regular HIV testing of breastfeeding mothers and need for ART-trained nurses to prescribe PrEP. Enablers included motivated and dedicated staff.

## Introduction

Vertical HIV transmission (VT) has declined significantly in sub-Saharan Africa in the last decade, with an estimated 76% reduction in South Africa, thanks to effective interventions such as the roll out of antiretrovirals (ARVs) to pregnant women living with HIV regardless of their CD4 counts (1–3). Despite this, South Africa still has one of the highest VT rates globally, estimated at 3% in 2022 (95% confidence interval [CI] = 2.6–3.2), resulting in a predicted 90,000 new paediatric infections over the next 10 years (2, 4, 5). Over one-third of VT can be attributed to incident HIV infection in mothers whilst pregnant or breastfeeding (5). It is estimated that pregnant and breastfeeding women (PBFW) are 3.6 times more likely to contract HIV than non-pregnant women (95% CI = 3.0–4.4); a recent prospective study identifying the postpartum period as the highest risk time for HIV acquisition (6, 7). This increased risk is linked to biological factors unique to these periods as well as behavioural factors (8–10). Over 1 million live births occur in South Africa with an estimated 700,000 from mothers not living with HIV (11). Primary HIV prevention in PBFW not living with HIV is urgently required to reach elimination of VT.

Although prevention of HIV is multipronged, one of the few effective female-controlled methods is the use of daily oral tenofovir-based pre-exposure prophylaxis (PrEP) (12, 13). A recent systematic review including 14 studies found PrEP exposure had no impact on pregnancy or perinatal outcomes (14, 15). Furthermore, very little PrEP is excreted in breastmilk (16). Based on this evidence, the World Health Organization (WHO) recognised that the benefit of taking PrEP for PBFW to prevent incident infections and VTs outweighs the potential risks for mother and child (17). In South Africa, modelling the impact of PrEP provision to PBFW shows a reduction in HIV incidence in PBFW between 48,000 and 136,000 averted new infections in the next ten years, resulting in a significant decrease in VT between 13.2% and 41.4%, depending on the scenario (4). In October 2021, the South African Department of Health (DOH) changed the PrEP guidelines to include provision of PrEP to PBFW at risk of HIV acquisition (18).

Studies in other sub-Saharan African contexts have demonstrated feasibility regarding PrEP implementation in maternal and child health (MCH) clinic settings (19). However, recent South African studies have showed that PBFW currently face several barriers to accessing PrEP, such as anticipated and internalized stigma, lack of provider knowledge, and challenges in disclosure of PrEP use (20, 21). Studies conducted across several different contexts have found that many health care providers (HCPs) describe their PrEP knowledge as insufficient, resulting in inaccurate patient education and HIV counselling, hesitancy to prescribe PrEP, and a lack of confidence in completing PrEP-related clinical activities (22, 23). Furthermore, studies conducted in sub-Saharan Africa found that many providers believed that oral PrEP may be inappropriate during pregnancy and for adolescent girls due to negative beliefs about adolescent sexuality and doubts that young women could handle the daily pill burden (23, 24). PrEP training among counsellors and healthcare providers has improved uptake and access to HIV counselling and testing, PrEP screening, uptake, and adherence (16, 22, 25, 26). Together these findings highlight the importance of practical provider PrEP training for the effective integration of PrEP into routine care for PBFW.

PrEP is a safe and effective intervention that, if adhered to, can prevent HIV acquisition in PBFW (14). Evidence suggests that integration of PrEP delivery into antenatal care (ANC) and postnatal care (PNC), by way of providers’ training and patient education, can increase and improve PrEP initiation and continuation in these populations (19, 25–27). However, despite these findings, there is a gap in the delivery of strategies and applying them to real-world conditions (28). The implementation science approach chosen for this study addresses this gap by applying the Reach Effectiveness-Adoption Implementation Maintenance (RE-AIM) framework to analyse the feasibility and acceptability of using provider PrEP training and mentorship to support the integration of PrEP into ANC and PNC services in eight clinics with high HIV prevalence within Cape Town, South Africa (29).

## Material and methods

### Study design and setting

Following changes in the DOH guidelines to include PrEP for PBFW, this implementation science study evaluated the acceptability, sustainability and feasibility of integrating PrEP for PBFW in eight primary care clinics in the Klipfontein and Mitchells Plain subdistricts, South Africa. These clinics are located in Cape Town high-density areas, have antenatal HIV prevalence ranging from 10 to 35% and were selected in consultation with the local district DOH based on client volume, geographical location and HIV prevalence (30). Community health centres (CHC) are larger clinics which include 24-hour Midwifery Obstetric Units (MOUs), defined as large antenatal facilities where women are referred to from smaller clinics (such as City of Cape Town [CCT] clinics) after 36 weeks of gestation to deliver. CHCs offer immunisation clinics 1–2 mornings per week compared to daily immunisation (“baby wellness”) clinics at CCT clinics. Past the first 6 weeks of postnatal care, there is no determined point for breastfeeding women (BFW) to access care, therefore, we aimed to find BFW in immunisation/ ”baby wellness” clinics.

The 2019 national antiretroviral (ART) guidelines recommend HIV testing for HIV-negative pregnant women at the initial ANC visit, at 20 weeks’ gestation, 32 weeks’ gestation and in labour. The same guidelines also recommend HIV testing for HIV negative BFW living every 3 months, starting at the 6 weeks postpartum visit until cessation of breastfeeding (31).

### Study population

#### Health care providers (HCPs)

Facility managers, counsellors, pharmacists, nurses and midwives who were offered DOH and supplementary University of Cape Town (UCT) training and consented to their data being analysed were included in the study (32). For the purpose of this study, we define as nurses: “professional nurses” (PN), “clinical nurse practitioner” (CNP) and we define as midwives: “midwives” and “advanced midwives”. We define as counsellors: “health promoters”, “lay counsellors”, “breast feeding counsellors”, while health care assistants include: “enrolled nurses” and “enrolled nurses assistants”. ART-trained nurses are defined as CNPs or professional nurses, midwives, advanced midwifes who have completed the Western Cape nurse-initiated and managed antiretroviral treatment (NiMART) programme (33). Provider data was collected from pre- and post-training questionnaires, including socio-demographic data (e.g., training, age and gender). Providers in a role where they could counsel or provide PrEP to PBFW were mentored by experienced UCT mentors on a regular basis (initially weekly then monthly, ranging from 2 to 5 times) and corresponding data was collected using assessment checklists (see Annex 1).

#### Pregnant and breastfeeding women

Following training and mentorship of HCPs in both ANC and “baby wellness” clinics, we monitored the offer, initiation and continuation of PrEP to eligible PBFW attending the clinic for their regular antenatal or “baby wellness” visits. Inclusion criteria included women eligible for PrEP as per the DOH PrEP guidelines and who did not opt out of aggregate data collection (34). Exclusion criteria entailed failure to meet the inclusion criteria.

#### Recruitment and enrolment

No direct recruitment was conducted for the study. In facilities, study staff collected de-identified aggregate data from HIV-negative PBFW eligible for PrEP. Providers at the selected facilities who participated in the PrEP training were asked to consent to have data from their pre- and post-training questionnaires and mentoring assessment collected. If they opted out of the study, they were still trained and mentored, however data was not collected.

#### Description of the implementation of the intervention

The intervention is described in Table 1.

#### Preparation

The implementation study staff was comprised of two research nurses, two counsellors, one data capturer and one medical doctor from UCT with experience implementing PrEP in PBFW within the region. First, the team assessed the readiness of the facility by assessing the antenatal HIV prevalence, the motivation of the facility manager, the number of ART-trained nurses available for providing PrEP, whether the facility had rolled out PrEP for other populations as well as the presence of other NGOs (non-governmental organisations) able to help with demand creation. If the clinic did not seem ready, we aimed to address existing structural issues with the local clinical governance officers and re-visited the facility three months later. Following the readiness assessment, the clinic manager identified the team to be trained, a date for training, and determined a designated PrEP champion at the clinic. The PrEP champion would be responsible for leading the implementation of PrEP at the clinic level, such as troubleshooting, reporting any PrEP-related issues to the implementation team, and following up on training.

#### Training

The implementation team advised the clinic team to complete the online DOH training prior to their training and then they trained HCPs (e.g., managers, midwives, nurses, counsellors and clerks) during a 2-hour didactic session (32). Trainees completed a pre-training assessment, consisting of 10 questions on their knowledge about PrEP safety and effectiveness, HIV prevention in pregnancy as well as completing a demographic questionnaire on their age, gender and previous trainings. After a brief introduction to PrEP, the team was divided into clinician and counselling groups for practical training. The clinical team received DOH-based PrEP training by the study nurse on PrEP initiation and continuation. In parallel, the study counsellor led a practical session for the counsellors on PrEP and daily adherence support. The clinical and counselling teams then regrouped to discuss any potential barriers in the clinic flow and found solutions together on how to address them. A post-training test was then completed by all trainees and assessed by the study doctor/nurse, with a pass score of 80% or higher. If a provider scored below 80%, the training information was reviewed individually between the HCP and the study team to ascertain if the provider or counsellor were ready to start PrEP counselling and provision. The clinic team was also given educational posters and job aid materials regarding PrEP in PBFW for the HCP, developed by DOH, as well as pamphlets on PrEP in PBFW, in English and isiXhosa, designed, developed and pre-tested by the study team at UCT.

#### Mentoring

The implementation team initially provided weekly mentoring to each clinic, wherein the study nurse and study counsellor only mentored trained nurses/midwives and counsellors who were working directly with PBFW. At each mentoring session, the implementation team completed an assessment checklist, scoring each provider on how well they were providing or counselling on PrEP (see Annex 1), based on the DOH PrEP training (35). The scores ranged from 0 (poor) to 10 (excellent), with feedback and comments provided at each assessment. This assessment allowed for measurement of PrEP integration feasibility by each provider within the clinic, defined as the degree to which the providers delivered PrEP counselling, initiation, and recording according to their training. As the mentored trainees progressed, the clinic mentoring frequency decreased to once per month. Each trainee received 2–5 mentoring visits in total, depending on operational needs.

#### Clinical folder review

In collaboration with the district clinical governance officers, we audited 10 folders of PBFW at each clinic to review quality of documentation and PrEP prescription. Results of the file review were communicated to the nurses and midwifes involved, as well as to their respective managers, and actions were taken by the clinical DOH staff to correct any errors, inconsistencies or missing data identified by the study team.

#### Handover

Six months after the initial training, the study team stopped mentoring and gave a detailed report to the clinic manager and the CCT or Provincial DOH team including the data collected on offer, initiation and continuation of PrEP among PBFW; results of the clinical folder reviews as well as results of problem-solving discussion with the staff on barriers encountered. Following the end of mentoring, the study team continued collecting data on the offer, initiation and continuation of PrEP in PBFW for at least 3 months.

#### Timelines

This implementation study started ran from February 2022 to July 2023, with continued post-handover monitoring through September 2023. Trained providers were mentored for 6 to 7 months.

#### Monitoring and evaluation

Information was collected from two sources: (1) aggregate data from facility logs on all PBFW attending regular ANC and “baby wellness” visits and (2) patient logs kept by HCPs on prescribing and counselling clients on PrEP. Information for antenatal pregnant women and for BFW were collected separately. The number of ANC visits gives an indication of the headcount and size of each clinic. It is, however, an overestimate for the MOUs as it includes women within the labour ward as well as women coming for their first postnatal visits. According to data collected from CCT, pregnant women tested for HIV on average 1.6 times per pregnancy in 2022, compared to the three times recommended in the DOH guidelines[1] (31). We therefore estimated the number of HIV-negative women eligible for PrEP by dividing the number of HIV negative tests done at each clinic by the number of average tests done per pregnancy. Because the number of BFW attending “baby wellness” clinics is not recorded, we used the number of babies attending the visits as a proxy.

The number of clinic attendees, HIV tests conducted, HIV-negative test results and women initiating and continuing PrEP was captured on a weekly basis by the study data capturer using standard clinic records and PrEP register. HIV antenatal prevalence was calculated by clinic and based on antenatal attendance and HIV prevalence at each clinic for the year 2021, as per the information supplied by the Provincial DOH. Accuracy of the data collected was triangulated for the CCT clinics by reviewing the CCT data on the monthly number of women attending ANC, having an HIV test with accompanying test results and initiating PrEP.

### Analysis

Using the RE-AIM framework for PrEP implementation work done among PBFW allowed us to analyse if the PrEP intervention was implemented successfully (28, 29). The application of the RE-AIM framework for the analysis of our data is summarised in Tables 1 and 2. The primary outcome was the number of health facilities trained and implementing PrEP, as per DOH guidelines, 3 months post end of mentoring (34). Secondary outcomes included the: (1) number of providers trained on providing PrEP and providing PrEP as per DOH guidelines; (2) number of PBFW who initiated PrEP; (3) number and proportion of PBFW who continued on PrEP (received a 3-months refill prescription).

### Ethics

#### Ethics approval

Ethical review and approval were provided by UCT Human Research Ethics Committee (UCT HREC) (ref 032/22). The intervention was approved by CCT Research Board (ref 9520) and by the Western Cape Province health Research (ref WC_202205_018).

#### Informed consent process

Passive consent was obtained at the clinics from women attending ANC and “baby wellness” visits with posters explaining the ongoing aggregate data collection about PrEP use and giving the option to women to opt out. Women who withdrew passive consent had the opportunity to withdraw consent. HCPs who participated in data collection (including pre- and post-training questionnaires, demographics, mentoring assessment) provided informed consent to participate in the study. All data was de-identified using de-linked patient identifiers. In the case of HCPS’ refusal to consent, they were still trained and mentored, however their data was not collected.

#### Reimbursement for Participation

No reimbursement was provided for participating women nor providers in this implementation science study.

## Results

### Reach, Effectiveness and maintenance

Overall 1493 women initiated PrEP out of 12,614 eligible HIV-negative women (12%). The two clinics (MOU 1 and MOU 3) with an HIV antenatal prevalence < 15% had lower proportion of women who started PrEP with 6% (168/2766) and 2% (83/5334), respectively. Clinic 1, Clinic 2 and MOU 2 (antenatal HIV prevalence around 30%) had higher PrEP initiation with 205/695 (29%), 147/661(22%) and 729/2283 (32%) respectively. The proportion who continued on PrEP (measured as PrEP refill at 1-month, 2-months and 3-months visits) ranged from 23% (39/168) to 70% (143/205) by clinic, with an overall continuation proportion of 41% (617/1493) among pregnant women in all clinics.

Among BFW, we evaluated PrEP initiation (or Reach) among women attending “baby wellness”/immunisation visits with their infants (Table 3). Because the number of BFW attending those visits is not recorded, the number of immunisation visits was applied as a proxy in estimating the size of each clinic. Overall, 34,230 immunisation visits were conducted during the study follow-up. Of their breastfeeding mothers attending the clinic, 1315 tested HIV-negative and 179 initiated PrEP (14% initiation). Of those, 45 (25%) continued PrEP and returned for refill at 1 month and 3 months. Across the 8 clinics, the proportion of BFW who initiated PrEP ranged from 2–28%. PrEP continuation at 3-months ranged from 0–59% by clinic and was 25% overall.

### Adoption

We evaluated the adoption at the staff level as fidelity to the PrEP guidelines. HCPs included mostly nurses and midwives but also a range of cadres from clerks to health assistants. Most counsellors, health assistants and clerks did not have post-secondary school qualifications. Despite recommending that all providers XXXcompleted the online DOH XXXPrEP training prior to the study training, only 18% had done so. 12% of HCPs had worked with PrEP before. A total of 42% of nurses and 37% of midwives were ART-trained (Table 5).

We describe the adoption of PrEP guidelines by HCP looking at the post-training test questionnaire results and mentoring scores [Additional file 2]. Nurses and midwives’ median post-test scores were 80% for non-ART trained cadres and 90% for those ART-trained. Counsellors’ post-test median score was 80%. Overall, 129 (58%) of HCPs had a post-training test score above 80% with admin clerks and health assistants having the least number of staff who passed the post-training test. The overall test results improved by 31% following training, with health assistant scores improving by 61% and counsellors by 34%. A median mentoring score at the last mentoring visit above 8 was observed among three-quarters of mentored cadres. Analysis of the mentoring assessments showed the following. For counsellors, mentoring helped them improve their assessment of the HIV risk profile of HIV-negative women, particularly assessment of exposure to intimate partner violence and alcohol or drug use. Mentoring of counsellors also helped them improve counselling on the importance of disclosing their PrEP use to others and establishing a plan to take PrEP daily. For nurses, mentoring helped improve skills related to counselling patients around potential PrEP side effects as well as ensuring the nurses gave women their next appointment dates, including the clinic’s phone number for any concerns or problems.

### Maintenance (provider and facility level)

**Figure 3.**
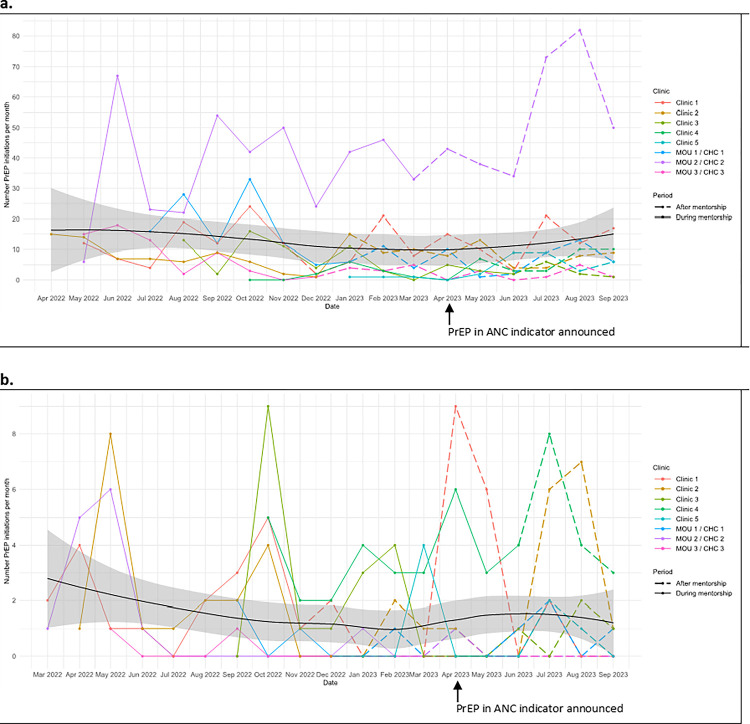
a. Number of pregnant women who initiated PrEP in each clinic during mentorship and after mentorship, with overall trend line across all clinics/MOUs. b. Number of breastfeeding women who initiated PrEP in each clinic during mentorship and after mentorship, with overall trend line across all clinics/MOUs.

Maintenance at the provider and facility level was measured by looking at the numbers of facilities still providing PrEP 6 months after the training date. All 8 clinics were still providing PrEP 6 months following training and 3 months following facility handover. In Table 6, we describe in greater detail clinic-specific events following handover. For pregnant women, there was an overall decrease in the number of monthly average PrEP initiations by 8% between the time the clinics were mentored and after the study mentorship ended. Some clinics (MOU2, clinic 2, clinic 4 and clinic 5) showed an increase in the number of monthly average PrEP initiations, with MOU2 having a much higher number of monthly PrEP initiations than other clinics. Three of the four clinics which showed a decrease in monthly average PrEP initiations (MOU1, MOU 3, clinic 1) had lower HIV antenatal prevalence (< 30%) than the others. When evaluating the average number of monthly PrEP initiations for each clinic over time (Fig. 3a), we saw that in October 2022, three clinics had an increase in PrEP initiations, while there were fewer PrEP initiations in five clinics from December 2022 to January 2023. From June to September 2023 (following mentorship period), there was an increase in PrEP monthly initiations in most clinics.

For BFW, the overall number of monthly average PrEP initiations mirrored that observed among pregnant women, decreasing by 4 (21%) after handover. Similarly, some clinics (MOU2, clinic 4 and clinic 5) showed an increase in their average PrEP initiations among BFW after handover. The average number of monthly PrEP initiations in BFW is represented in Fig. 3b. In October 2022, there was also an increase in PrEP initiations in four clinics and a decrease in December 2022 in 5 clinics, while in June and July 2023 there was an increase in PrEP initiations in four clinics.

## Discussion

Integration of PrEP into ante- and postnatal care facilities was acceptable to PBFW and feasible among trained providers. Despite overall acceptability, PrEP initiation and continuation in PBBW were lower than expected from our clinical trial, PrEP-PP (15). Adoption and implementation at the provider level was high for nurses and midwives, but lower in counsellors, indicating the need for continued training and support in those cadres. All eight facilities continued providing PrEP post-handover with a slight overall decrease in PrEP initiation among PBFW over time.

### Acceptability among pregnant women

PrEP counselling and delivery was well integrated into the ANC cascade for both small and larger antenatal clinics with HIV-negative pregnant women undergoing recommended HIV testing alongside the offer of PrEP from counsellors (31). Those interested then initiate PrEP with the midwife or nurse during their normal antenatal visit. Bloods are taken, with other bloods required for standard ANC, and oral PrEP is dispensed in the room, a flow resembling the one-stop-shop model recommended for better patient satisfaction (35). This easy integration of PrEP delivery into ANC built upon the strong integration of VTP in this setting and was most efficient in clinics with the highest HIV prevalence. Despite this successful integration, PrEP uptake was lower than expected compared to our parent clinical trial study PrEP-PP implemented in a similar setting, where roughly three quarters of pregnant women approached were interested in starting PrEP (15).

Clinics in higher HIV prevalence communities (> 20% antenatal prevalence) had higher levels of PrEP acceptability and continuation compared to clinics in lower HIV prevalence communities among PBFW. This could be due to a variety of factors, including different risk perception and HIV-related stigma (36). We also observed an increase in PrEP initiations in September–October 2022 in 4 out of 6 clinics and a decrease in 6 out of 8 clinics in December 2022–January 2023, suggestive of external factors. Surges in initiations and testing could be attributed to the pre-festive season with decreased proportion of HIV testing and PrEP initiation during the festive season, as documented elsewhere in South Africa (37). This implementation science study was conducted in a real-world setting with no dedicated study staff providing counselling, PrEP prescriptions or financial compensation for PBFW, as would be provided in a randomised controlled trial design. As such, our results are similar to a real-world implementation program conducted in Kenya where PrEP delivery was integrated into 16 antenatal clinics and 18.6% of pregnant women were initiated on PrEP (of n = 4912 tested HIV-negative) (38). In the PrIYA study, dedicated program nurses offered PrEP counselling, whereas in our study, DOH-affiliated staff were trained and mentored and remained the only individuals counselling and prescribing PrEP throughout.

Reasons for our comparatively lower acceptability could include stigma faced in certain communities, low risk perception from women at substantial risk (for example with a partner of unknown HIV status), concerns around safety of PrEP in pregnancy as well as sexual activity declining in late pregnancy compared to early pregnancy (27, 28, 39, 40). Possible health-system barriers described in the literature include the additional work burden from PrEP-specific activities, PrEP hesitancy linked to inadequate HCP training, inappropriate PrEP beliefs stemming from lack of PrEP knowledge in HCPs, concerns around PrEP safety in pregnancy as well as the need for specific monitoring of PrEP initiation in PBFW (23, 41, 42). Some of the health system barriers observed on the ground, explored within forthcoming complimentary qualitative research on PrEP integration from the HCP point of view, were shortage and high turnover of staff and requirements for nurses prescribing PrEP to be ART-trained (43). ART-trained nurses are themselves in low numbers, overburdened and not necessarily in services where PrEP can be integrated with other services (44).

### Acceptability among breastfeeding women

There was limited integration of maternal and child health, apart from the 6 weeks immunisation visit when testing of HIV-negative breastfeeding mothers occurred. The 3-monthly testing whilst breastfeeding was very poorly implemented in most of the mentored clinics, with very little subsequent maternal and child integration. Despite postnatal transmission being a significant driver of VT, testing of BFW remains neglected in South Africa (7, 45, 46). Therefore, in our study, the denominator of BFW testing for HIV was very low at < 2000 women and only 179 (14%) of which started PrEP. In comparison, within the Kenyan PrIYA study, 25% initiated PrEP of n = 4467 women testing HIV-negative (38). Possible reasons for the lower HIV testing include limited integration of maternal and child services, despite international and national recommendations (41, 47, 48). However, after workshopping potential solutions to the identified barriers with clinic managers and staff, we saw the number of PrEP initiations increase among BFW. This could also be attributed to an increase in awareness of PrEP being recommended for PBFW over time. Other barriers to PrEP initiation in BFW were similar to barriers described among pregnant women, with the lack of ART-trained nurses in child services being particularly problematic. With the release of new DOH VTP guidelines calling for integration of maternal and child services postpartum, we hope that fixed testing timepoints for BFW (10 weeks, 6 months and then 3 monthly) will increase HIV testing and the subsequent offer and counselling about the benefits of PrEP use in this period. (47)

### Effectiveness and maintenance (patient level)

Continuation was slightly higher among pregnant women than BF women, and was comparative to the PrIYA study participants at the 3-month period (38, 41). Lower persistence on PrEP could be partly explained by the fact that, PrEP initiation and continuation relied solely on DOH ART trained nurses and counsellors, who may be overstretched with their work with women living with HIV (WLHIV). Furthermore, PrEP continuation is likely underestimated as it only looks at continuation for women going back to the same clinic. If the woman went to a different clinic for her PrEP continuation, or if she had to continue her postpartum care in a different clinic than the delivery clinic, it would not be captured in our data. In South Africa, pregnant women are seen at least four times whilst pregnant, and high proportions of PrEP continuation are likely linked to frequent antenatal visits (48). For BFW, because of the lack of integration of maternal and child services, their point of care is not well defined. They could be seen as a dyad with their infant or alternatively if they come for contraception under sexual and reproductive health (SRH) care.

Some of the patient-related barriers to PrEP continuation identified in the literature are linked to pill fatigue, PrEP side effects, stigma, lack of disclosure, fear of partner violence and reduction in sexual activity (49–52). For BFW, reasons for PrEP discontinuation are thought to be similar to reasons behind postpartum disengagement from ART and include the demands of motherhood as well as the perception that the infant’s HIV risk decreases post pregnancy (53–57). Some of the health system barriers identified include difficulty accessing care (for transport and financial reasons), lack of PrEP availability at certain clinics, as well as lack of PrEP knowledge from HCPs (49). We found very little research on health system barriers to PrEP continuation in only BFW (49–52). Some of the barriers to continuing PrEP we observed on the ground were related to the lack of horizontal and vertical integration in the implementation of PrEP in PBFW. There were very few primary care clinics in the surrounding areas providing PrEP for PBFW (other than the eight we trained), which meant that once women had delivered their babies, they often struggled to find a clinic to continue PrEP. Finally, within a same clinic, PrEP was not readily offered in other services such as family planning. These challenges emphasize the need for future research focusing on understanding unique health system barriers and facilitators to PrEP initiation and continuation among PBFW, especially for those postpartum.

### Adoption and maintenance (provider level)

There was good buy-in from participating clinics and adequate support from the local health authorities. The proportion of trained providers who counselled and provided PrEP according to the training and to the DOH guidelines was good. Although mentorship was only provided to nurses and counsellors actively counselling and providing PrEP to PBFW, we trained a much larger portion of clinic staff, with the aim of increasing PrEP knowledge in the community and in cadres who are frontline workers, providing informal health promotion. Furthermore, by highlighting the evidence on PrEP safety in PBFW and increasing PrEP awareness among HCPs, we hoped to increase their confidence and ability to better implement PrEP, as found by other colleagues (58). We found that the staff was eager to learn about PrEP. Although the pre-training tests results were often quite low, confirming that HCP PrEP knowledge was poor prior to the training the post-training questionnaires increased by a median of 31% (23). Training and mentoring of HCP on PrEP in PBFW had a positive effect on the overall delivery of PrEP in the facilities with a reported overall increase in PrEP initiation in the CCT clinics[2]. Our findings reinforce that of other studies, showing that training HCP on PrEP improved HIV counselling, PrEP screening, uptake and adherence (42, 58, 26). Only 18% of the trained HCP had previously completed the suggested online DOH training, demonstrating that additional research is needed to understand the best ways in which to offer PrEP training to HCP to maximize reach.

Although in our research we only included the eight clinics where we were providing the implementation science study, we found that, at the time of our study, the implementation of PrEP for PBFW had not started in other clinics in the same sub-district in Cape Town[3] (34). As mentioned above, this affected the PrEP continuation of PFBW as it limited the access of BFW to PrEP. Effective implementation of new guidelines often comes with new monitoring tools, such as national indicators (28). In April 2023, the DOH announced new national indicators, including indicators tracking PrEP initiation and continuation in ANC. The implementation of these new indicators may have contributed to the increases observed in PrEP initiation between June -September 2023. However, there is still no indicator for BFW and this could negatively impact both PrEP initiation and continuation.

All of the study clinics were still delivering PrEP to PBFW more than three months after handover, showing that PrEP delivery is sustainable. The overall mean monthly number of PrEP initiations in pregnancy whilst mentoring was 85 and declined to 78 without our regular study mentoring visits. A decline in number of initiations once mentoring support is removed is to be expected (26). It was encouraging to see that the overall number of monthly PrEP initiations in BFW decreased by only 4 (21% ) after handover. Furthermore, we also observed an increase in PrEP initiations in PBFW in June -July 2023 in 4 clinics. Clinics 2, 3 and 4 had a sustained increase in their BFW PrEP initiations from June to September 2023. Many factors could have played a role. Firstly, it is likely that community PrEP knowledge increased during the time of our implementation, resulting in an increase in PrEP demand and uptake (60–62). Secondly, during the timing of our implementation, we worked in partnership with the local health authorities and highlighted some of the difficulties we encountered on the ground. This led to workshops with the clinics, with the support of the local authorities and led to some significant changes in the clinics’ flow, particularly for BFW. For example, one clinic decided to test all mothers at the 14-week immunisation visit to facilitate offering of PrEP, on top of the normal 6 weeks testing. Another clinic implemented an appointment system for the mother and babies at 3-monthly interval to improve maternal HIV testing. These changes could have resulted in improvement in HIV testing and PrEP offer to BFW and outbalanced the likely decrease in PrEP initiation due to our team not supporting anymore.

### Strengths and limitations

The key strength is the real-world implementation of the study with limited study involvement, without additional PrEP dedicated staff than a very small training and mentoring UCT team working in collaboration with DOH staff. We used real-world program data which is crucial to understand implementation. This increases the generalisability of our study findings to other similar settings.

Limitations include reduced capacity for collection of additional data beyond routine aggregated data collected by DOH staff. For example, although we could identify how many pregnant women were tested for HIV, it was very difficult to ascertain how many of them were offered PrEP. It was also difficult to ascertain how many BFW accompanied their babies to their immunization visits and were tested for HIV as no indicators are collected. This highlighted the need to implement new indicators to be able to follow BFW for improved PrEP implementation and ultimately curb postnatal HIV transmission.

## Conclusion

PrEP implementation in ANC and services for BFW was feasible, acceptable and sustainable. PrEP initiation and continuation among PBFW was lower than expected but improved over time, particularly after the implementation of new national monitoring indicators. Barriers to the integration of PrEP, particularly for breastfeeding women, were observed and included limited, opt-in repeat HIV testing of breastfeeding mothers and need for ART-trained nurses to prescribe PrEP. Enablers included motivated and dedicated staff.

## Figures and Tables

**Figure 1:**
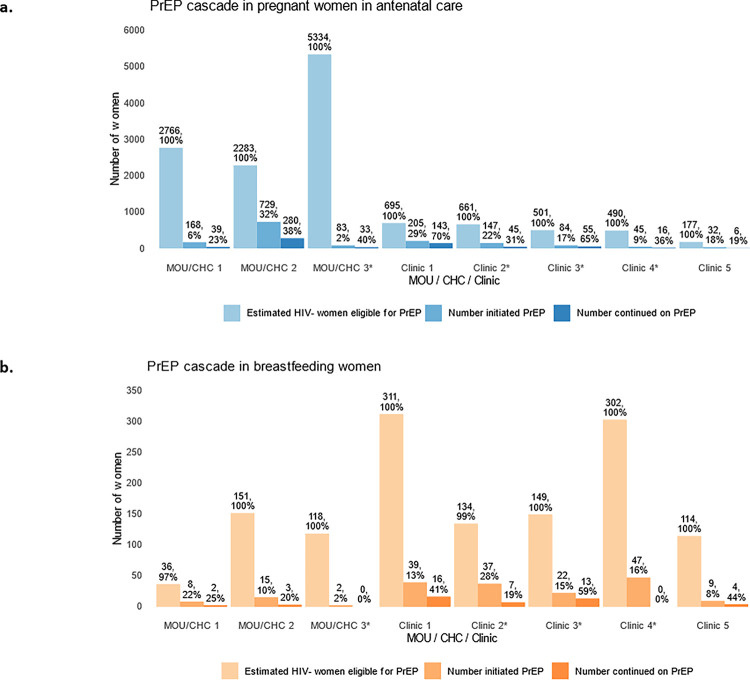
PrEP initiation and continuation cascade in a) pregnant women in antenatal care and b) breastfeeding women in 8 clinics (March 2022– September 2023) *Facilities mentored for more than 7 months.

**Table 1:** Description of the intervention from determinants to outcome

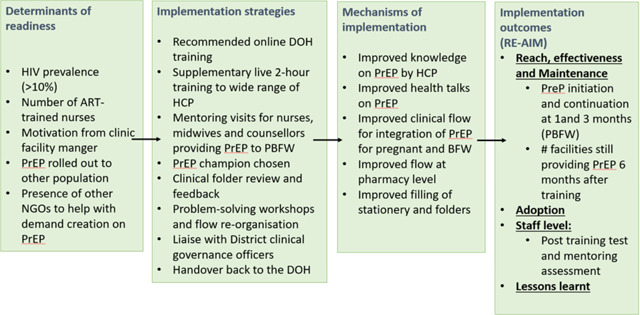

ART: Antiretroviral therapy; DOH: department of Health, HCP: Healthcare practitioner; NGOS: Non-Governmental Organizations; PBFW: pregnant and breastfeeding women; PrEP: Pre-exposure prophylaxis

**Table 2:** Study Outcomes mapped using the RE-AIM Framework

RE-Aim Framework	Outcomes	Measurement	Source	Frequency
Reach	PrEP initiation (acceptability)	Numerator: # of women from denominator who start PrEP. Stratified by pregnant vs postpartum.	Clinic routine data: counsellor’s HIV testing book, clinic attendance record, PMTCT register, electronic clinic indicators, PrEP Department of Health register, PrEP register	Completed by counsellor and nurses and captured weekly by data capturer.
Effectiveness and Maintenance (Individual and facility level)	PrEP continuation (sustainability)	# facilities providing PrEP 3 months post end of mentoring % of women returning within 3 months for a PrEP refill prescription;	PrEP Department of Health register PrEP register	PrEP register – as patient is seen – to be completed by providers during the refill visit. Captured weekly by data capturer.
Adoption (Staff level) and Implementation	Adherence to PrEP protocol (feasibility)	Feasibility is defined as the proportion of trained providers who provide PrEP services according to training and standard operating procedures.	- Pre and post-training test - PrEP mentoring assessment checklist – completed by the study nurse/counsellor	- Pre and post-test questionnaire completed by trained health care provider at the start and end of each training -Mentoring assessment filled weekly or when nurse/counsellor is offering PrEP to a client.

**Table 3:** Overall flow of PrEP cascade in pregnant women in antenatal care in 8 clinics (March 2022- September 2023)

Clinic	HIV antenatal prevalence (2021)	# of ANC visits*	# of HIV tests in pregnant women	# of HIV negative tests in pregnant women	Estimated # HIV-negative pregnant women eligible for PrEP**	# and % PrEP initiated (of HIV-tested)	# and % continued at 1m and 3m
MOU/CHC 1	13%	18 947	4 477	4 425	2 766	168 (6%)	39 (23%)
MOU/CHC 2	34%	20 534	3 763	3 653	2 283	729 (32%)	280 (38%)
MOU/CHC 3	10%	24 610	8 603	8 534	5 334	83 (2%)	33 (40%)
Clinic 1	28%	2 378	1 121	1 112	695	205 (29%)	143 (70%)
Clinic 2	35%	1 461	1 072	1 057	661	147 (22%)	45 (31%)
Clinic 3	38%	1 244	816	802	501	84 (17%)	55 (65%)
Clinic 4	24%	1 032	794	784	490	45 (9%)	16 (36%)
Clinic 5	33%	398	290	283	177	32 (18%)	6 (19%)
**Total**		**69 752**	**20 466**	**20 183**	**12 614**	**1 493 (12%)**	**617 (41%)**

* MOU headcounts may include postpartum women attending for the initial postnatal visit and women in labour

** Estimates of 1.6 HIV tests conducted in antenatal care per CT Metro data from 2022

**Table 4:** Overall flow of PrEP cascade in breastfeeding women in care in 8 clinics (March 2022–July 2023)

Clinic	HIV antenatal prevalence (2021)	Number of immunization visits per clinic*	Number of breastfeeding mother tested & counseled for HIV	Estimated number of HIV-negative mothers eligible for PrEP**	Number and percentage of PrEP initiations	Number and percentage of PrEP refill
MOU/CHC 1	13%	574	37	36	8 (22%)	2 (25%)
MOU/CHC 2	34%	271	151	151	15 (10%)	3 (20%)
MOU/CHC 3	10%	415	118	118	2 (2%)	0 (0%)
Clinic 1	28%	10 040	311	311	39 (13%)	16 41%)
Clinic 2	35%	12 847	135	134	37 (28%)	7 (19%)
Clinic 3	38%	4 905	149	149	22 (15%)	13 (59%)
Clinic 4	24%	1 567	302	302	47 (16%)	0 (0%)
Clinic 5	33%	3 701	114	114	9 (8%)	4 (44%)
**Total**		**34 320**	**1 317**	**1 315**	**179 (14%)**	**45 (25%)**

* Number of BF women attending is unknown, the number of immunization visits is used as a proxy

** Average number of tests for breastfeeding women is 1

**Table 5:** Demographics of HCP completing the PrEP training

	Overall	Nurses	Midwives	Counsellor	Health assistants	Admin clerk	Clinic manager	Other HCW*
	N (%)	n (%)	n (%)	n (%)	n (%)	n (%)	n (%)	n (%)
Total	224	108 (48%)	19 (8%)	37 (17%)	33 (15%)	16 (7%)	8 (4%)	3 (1%)
Post seconday school qualifications	177 (79%)	108 (100%)	19 (100%)	3 (8%)	33 (100%)	3 (19%)	8 (100%)	3 (100%)
Years of experience (median, IQR)	7 (4–11)	5 (3–10)	10 (7–15)	9 (7–10)	10 (5–13)	7 (5–11)	10 (7–18)	8 (6–9)
Worked with PrEP before	27 (12%)	18 (17%)	1 (5.6%)	6 (17%)	0 (0%)	0 (0%)	2 (29%)	0 (0%)
DOH PrEP training done (online)	39 (18%)	20 (19%)	2 (11%)	14 (42%)	1 (3%)	0 (0%)	2 (29%)	0 (0%)
ART-trained	52 (23%)	45 (42%)	7 (37%)	-	-	-	-	-

* Other HCWs include 2 physicians and 1 pharmacist

**Table 6:** Maintenance of prescribing PrEP at the clinic level comparing the mean PrEP prescriptions whilst clinic was mentored to after the clinic was being handed over

				Pregnancy			Breast feeding		
Clinic	Dates of staff mentoring	Months mentored	Antenatal HIV prevalence	% Change	1. Average PrEP initiations per month during mentorship	2. Average monthly PrEP initiations in three months after handover	% Change	3. Average PrEP initiations per month during mentorship	4. Average PrEP initiations in three months after handover
Clinic 3	Aug 2022 – Jun 2023	10	38%	53% ↓	6	3	50% ↓	2	1
Clinic 2	Apr 2022 – Jan 2023	9	35%	10% ↑	8	9	30% ↓	2	1
Clinic 4	Sep 2022 – Jun 2023	9	33%	118% ↑	2	5	41% ↑	4	5
MOU/ CHC 3	Apr 2022 – Dec 2022	8	10%	48% ↓	8	4	No change	0	0
MOU/ CHC 2	Aug 2022 – Mar 2023	7	34%	3% ↑	37	38	69% ↑	1	0
Clinic 1	Apr 2022 – Nov 2022	7	28%	25% ↓	13	10	68% ↓	2	1
Clinic 5	Nov 2022 – Jun 2023	7	24%	157% ↑	2	6	No change	1	1
MOU1 /CHC 1	Jun 2022 – Jan 2023	6	13%	65% ↓	8	3	44% ↓	1	0
**Total**				**8%**↓	**85**	**78**	**21%**↓	**13**	**9**

## Data Availability

The data that support the findings of this study are available from University of Cape Town but restrictions apply to the availability of these data, which were used under license for the current study, and so are not publicly available. Data are however available from the authors upon reasonable request and with permission of University of Cape Town

## List Of Abbreviations

ANC: Antenatal Care
ART: Antiretroviral Therapy
BFW: Breastfeeding Women
CHC: Community Health Centre
CCT: City of Cape Town
CI: Confidence Interval
CNP: Clinical Nurse Practitioner
DOH: Department of Health
HCP: Healthcare Practioner
HIV: Human Immunodeficiency Virus
MCH: Maternal and Child Health
MHS: Metro Health Services
MOU: Midwifery Obstetric Unit
NGO: Non-Governmental Organisations
NIMART: Nurse Initiation and Management of ART
PN: Professional Nurse
PNC: Postnatal Care
PrEP: Pre-exposure prophylaxis
RE-AIM: Reach Effectiveness Adoption Implementation and Maintenance
UCT: University of Cape Town
VT: Vertical Transmission
WHO: World Health Organization
WLHIV: Women Living with HIV
